# ID 134 - Estudo de Custo da Doença da Histoplasmose Disseminada em Pessoas Vivendo com HIV/Aids

**DOI:** 10.5327/2237-9622.2025.v34s1.134

**Published:** 2025-11-25

**Authors:** Raissa Allan Santos Domingues, Maria Regina Fernandes de Oliveira

**Keywords:** análise de custo de doença, diagnóstico, histoplasmose disseminada, HIV

## Abstract

**Introdução:**

A histoplasmose é uma micose sistêmica causada por fungos dimórficos da espécie . A histoplasmose disseminada é uma das principais infecções que ajuda a definir a aids e uma das principais causas de morte de indivíduos com HIV, no Brasil e nas Américas. Por não ser uma doença de notificação compulsória, é considerada negligenciada nos países endêmicos, inclusive no Brasil. A semelhança clínica com outras infecções oportunistas, a variação da sintomatologia e diferentes respostas à terapia, em indivíduos imunocomprometidos infectados por, tornam o diagnóstico correto e precoce fundamental, viabilizando o tratamento específico que tem como intuito reduzir o risco de complicações e diminuir a elevada letalidade associada à doença disseminada. Até o momento não foram realizadas avaliações econômicas para a detecção do antígeno de histoplasma no Brasil, como integrante do fluxo de diagnóstico preconizado. O presente estudo tem o objetivo de relacionar os custos gastos com a doença para subsidiar a tomada de decisão para a incorporação dos testes de detecção do antígeno de a fim de diagnosticar a infecção por histoplasmose na rede de saúde do SUS em pessoas vivendo com HIV/aids (PVHA).

**Método:**

O estudo de custos da histoplasmose disseminada foi realizado nas perspectivas do SUS e da sociedade; para isso, foi organizada uma coorte baseada em dados epidemiológicos de casos notificados de HIV e de histoplasmose no Brasil no ano de 2021. Foram estimados os custos diretos médicos relacionados ao diagnóstico atualmente disponível no SUS: cultura de células, exame direto e teste de imunodifusão, aos medicamentos preconizados no protocolo clínico (anfotericina B e itraconazol), à assistência e ao tratamento ambulatorial e hospitalar dos casos de histoplasmose disseminada, além dos casos de reinfecção/reativação; e os custos indiretos, relacionados à perda de produtividade por mortalidade precoce e por morbidade analisados pelo método do capital humano. Foram realizadas análises de sensibilidade bivariada determinística variando o número de pacientes na coorte, o preço dos medicamentos e os exames praticados no Brasil, além da frequência de consultas e exames, conforme protocolo clínico.

**Resultados:**

O custo total da histoplasmose no Brasil no ano de 2021 foi de R$ 144.073.776,03 (82.036.345,98 – 672.725.717,89) e a maioria foi relacionada aos custos indiretos devido à mortalidade precoce e perda de produtividade por morbidade (58,54%), seguido dos custos com medicamentos e tratamento hospitalar dos casos novos (28,84%) e do tratamento hospitalar em casos de reinfecção/reativação (5,59%), custo dos procedimentos com finalidade diagnóstica (3,67%), e por fim, o custo do tratamento ambulatorial para profilaxia secundária e exames de monitoramento (3,37%).

**Conclusão:**

Ponderando que estudos econômicos não são, geralmente, generalizáveis e não é adequado extrapolar avaliações econômicas de outros países, esse estudo é necessário, por ser uma avaliação específica para a realidade brasileira, abordando as peculiaridades do SUS, do comportamento da doença entre os brasileiros e dos custos de diagnóstico, tratamento e cuidado aos pacientes no País. A análise proposta poderá subsidiar o processo de decisão para ampliar o acesso diagnóstico aos casos suspeitos de histoplasmose disseminada, necessário para propiciar o tratamento adequado e promover a assistência à saúde de qualidade às PVHA no Brasil, além de reduzir os óbitos relacionados à doença.

